# Comparative genomic analysis of five Eimeria spp. in rabbits provides insights into coccidian tissue tropism

**DOI:** 10.1099/mgen.0.001576

**Published:** 2025-11-27

**Authors:** Tianyi Hou, Dongle Su, Xinran Wang, Yanhua Xu, Junhong Lu, Qi Wang, Tianpeng Wang, Rui Xu, Yaqiong Guo, Na Li, Xun Suo, Yaoyu Feng, Lihua Xiao

**Affiliations:** 1 State Key Laboratory for Animal Disease Control and Prevention, Center for Emerging and Zoonotic Diseases, College of Veterinary Medicine, South China Agricultural University, Guangzhou 510642, PR China; 2 College of Veterinary Medicine, China Agricultural University, Beijing 100083, PR China

**Keywords:** comparative genomics, *Eimeria*, invasion mechanism, rabbits, surface antigens, tissue tropism

## Abstract

*Eimeria* spp. are common coccidian parasites of a wide range of vertebrates, causing diarrhoea, poor weight gain and significant mortality in domestic animals and birds. However, there is a paucity of genomic data on these important pathogens. Of the 11 common *Eimeria* species in rabbits, only *Eimeria stiedae* invades biliary epithelial cells rather than the intestine, and the determinants of coccidian tissue tropism remain unclear. In this study, we sequenced the genomes of five common rabbit *Eimeria* species, including *E. stiedae*, *Eimeria flavescens*, *Eimeria intestinalis*, *Eimeria magna* and *Eimeria media*. Comparative genomic analysis reveals that the genome of *E. stiedae* appears more compact than the genomes of intestinal *Eimeria* species. It shows reductions in the numbers of rhoptry proteins, dense granule proteins, microneme adhesive repeats and TA4 surface antigens, suggesting that surface and invasion-associated proteins may be involved in the tissue tropism of *Eimeria* spp. In addition, *E. stiedae*-specific motifs are identified in a cluster of hypothetical surface antigens. These data provide not only new insights into the biological characteristics of coccidia but also valuable resources for functional research and drug and vaccine development.

**Impact Statement**

Coccidiosis caused by *Eimeria* spp. leads to significant economic losses in the rabbit industry, yet the mechanisms governing tissue tropism of different species remain unclear. Our comparative genomic analyses of five rabbit *Eimeria* species have revealed key differences in invasion-associated proteins between hepatic (*Eimeria stiedae*) and intestinal species, shedding light on the putative molecular basis of tissue tropism. These findings advance our understanding of coccidian biology and provide critical targets for developing novel drugs or vaccines to combat coccidiosis in livestock and humans.

## Data Summary

The whole-genome sequencing data of all strains in this study have been submitted to the National Center for Biotechnology Information (NCBI) database under the BioProject accession number PRJNA1277586. The Sequence Read Archive (SRA) under accessions SRR34002152–SRR34002158 is publicly accessible at https://www.ncbi.nlm.nih.gov/sra/. The genomes with complete annotations are publicly accessible at https://www.ncbi.nlm.nih.gov/genbank/ under GenBank accession JBPVOE000000000, JBPVOF000000000, JBPVOG000000000, JBPVOH000000000 and JBPSDH000000000. The accession numbers for the apicoplast genome sequences are JBPVOE010002295.1, JBPVOG010000241.1, JBPVOF010000045.1, JBPVOH010000182.1 and JBPSDH010000013.1, while the accession numbers for the mitochondrial genome sequences are JBPVOE010002751.1, JBPVOF010000019.1, JBPVOG010000174.1, JBPVOH010000171.1 and JBPSDH010000772.1. The data and code used in the analysis are available at GitHub through the following link: https://github.com/tyhou/Eimeria.Comparative.Genomic.

## Introduction

Coccidiosis caused by *Eimeria* spp. is responsible for high morbidity, mortality and poor growth in livestock and poultry, resulting in significant economic losses to agriculture worldwide [1]. Rabbits are particularly susceptible to coccidiosis. Of the 11 *Eimeria* species in rabbits, *Eimeria stiedae* is the most pathogenic species and the only one that develops in the epithelium of the bile duct and causes hepatic coccidiosis [2]. The remaining species parasitize different parts of the intestine and cause intestinal coccidiosis of varying severity [3,4]. However, the genetic determinants of coccidian tissue tropism are poorly understood. This is largely due to the lack of whole-genome sequence (WGS) data from *Eimeria* species, which has hindered the identification of key genetic factors. Although a CRISPR/Cas9 system has been established for *Eimeria tenella* and has been applied to functional gene studies [5,6], genomic data for rabbit coccidia are still lacking. To date, only the genomes of *Eimeria falciformis* from mice and the seven *Eimeria* species from chickens have been sequenced [7,9].

Tissue tropism is manifested by parasite recognition and colonization of specific host cells during invasion. Invasion of host cells by apicomplexans is a highly regulated process involving the secretion of multiple secretory organelles, including micronemes, rhoptries and dense granules [10]. During the pre-invasion period, calcium ions stored in the endoplasmic reticulum of the parasite are released upon stimulation by a signal, causing an increase in cytoplasmic calcium levels. Calcium-dependent kinases are activated to regulate microneme secretion and kinesin complex formation on the inner membrane complex [11,12].

Many secretory proteins contribute to the tissue tropism of coccidia. Among them, micronemal proteins (MIC) have an adhesive function and interact with host cell membrane receptors during invasion [13]. Since *Neospora caninum* and *E. tenella* have a smaller MIC repertoire than *Toxoplasma gondii*, it is thought that these proteins are involved in tissue tropism [14]. Recent substitution studies of microneme adhesive repeat (MAR) domains support this idea and suggest that these proteins play a critical role in tissue tropism in *Eimeria acervulina*, *Eimeria maxima* and *Eimeria mitis* [15]. The rhoptry releases the rhoptry neck (RON) and bulb (ROP) proteins at the pre- and post-invasion stages, respectively [16]. RONs then bind to the apical membrane antigen 1 [17,18] and participate in forming the moving junction (MJ) between the host and parasite cell membranes. A previous study showed that *Tg*ROP18 interacts with host cell vimentin, which is minimally expressed in brain tissue. Therefore, ROP proteins are also associated with tropism [19]. In *T. gondii*, dense granule proteins (GRA) are secreted into the parasitophorous vacuole (PV) as well as the host cytosol and nucleus [20], contributing to PV and PV membrane (PVM) modification. Additionally, surface antigens (SAG) can bind to polysaccharide receptors on the host cell surface, contributing to parasite tissue tropism. Due to the lack of WGS data, very little is known about surface and invasion-associated proteins in rabbit *Eimeria* spp., despite significant differences in tissue tropism among them.

In this study, we sequenced the genomes of one hepatic species (*E. stiedae*) and four intestinal species (*E. flavescens*, *E. intestinalis*, *E. magna* and *E. media*) of *Eimeria* from rabbits. This finding suggests that the genetic signatures could be associated with their distinct tissue tropisms. Comparative genomic analysis demonstrated that *E. stiedae* has a compact genome. Some invasion-related proteins have lower copy numbers in *E. stiedae* than in intestinal *Eimeria* species. Notably, *E. stiedae* lacks some conserved amino acids in TA4 surface proteins and possesses a cluster of hypothetical surface glycoproteins (RbE-SAGs) with a unique motif. These data provide new insights into the possible cause of the tissue tropism of *Eimeria* spp. and are a valuable resource for biological studies of coccidia.

## Methods

### Parasite isolates and whole-genome sequencing

The *Eimeria* species used for whole-genome sequencing (including *E. stiedae*, *E. flavescens*, *E. intestinalis*, *E. magna* and *E. media*) were isolated from experimentally infected rabbits in Beijing, China. *Eimeria* species identification was based on oocyst size and morphology as previously described [21]. These *Eimeria* isolates were established by experimental infection of specific pathogen-free New Zealand White rabbits with individual sporulated oocysts. Saturated salt flotation was used to purify oocysts from faecal samples collected from rabbits infected with each *Eimeria* species [22]. This research was approved by the Committee on the Ethical Use of Animals in Research, South China Agricultural University (No. 2021C051).

DNA was extracted from the purified oocysts after five freeze-thaw cycles using the QIAamp DNA mini kit (Qiagen Sciences, Hilden, Germany). The DNA preparations were used for library construction and sequencing on an Illumina HiSeq 2500 (Illumina, San Diego, CA, USA) using the 250 bp paired-end technique. WGS data of other apicomplexans used in this study were downloaded from ToxoDB (http://ToxoDB.org) and the National Center for Biotechnology Information (NCBI; https://www.ncbi.nlm.nih.gov/).

Transcriptomic data were obtained from sporulated and unsporulated oocysts of *E. stiedae* by RNA sequencing. RNA extraction, library construction and transcriptome sequencing of the oocysts were performed by Guangzhou Gene Denovo Biotechnology Co. Ltd. using the Illumina 150 bp paired-end approach.

### Genome assembly

The quality of the raw sequencing data was checked using FastQC v0.11.05. Clean reads were obtained by trimming adapter sequences and low-quality reads (below Q30) with default parameters using Trimmomatic v0.39 and assembled *de novo* using CLC Genomics Workbench v12.0.3 with a word size of 63 and a bubble size of 400. In addition, genomes were assembled using SPAdes v3.1 in careful mode. Contaminating sequences were identified and removed using blast+ v2.7.1 [23] against the NCBI core nucleotide database with an e-value threshold of 1e^−10^. After the removal of contaminating sequences, the draft genomes of five rabbit *Eimeria* species were obtained. Genome assembly statistics were generated using QUAST v5.2.0 [24]. The identity of the five rabbit *Eimeria* species was confirmed based on blast analyses of the 18S rRNA (GenBank accessions: HQ173830, HQ173831, HQ173833, HQ173834 and HQ173837) and ITS-1 (GenBank accessions: HM768883, HM768884, HM768886, HM768887 and HM768890) sequences extracted from the genome assemblies.

### Gene prediction and annotation

Reads from the transcriptome sequencing of sporulated and unsporulated oocysts of *E. stiedae* were mapped to the genome using TopHat v2.0.14 and Cufflinks v2.2.1 [25]. The cDNA sequences and gene structure annotations were generated using PASApipeline (Program to Assemble Spliced Alignments) (https://github.com/PASApipeline/PASApipeline). These data were converted to GenBank format using the built-in script ‘gff2gbSmallDNA.pl’ and served as a training model for gene prediction using AUGUSTUS v3.3.2 [26]. In addition, Geneid v1.4.5 [27] and GeneMark-ES [28] were used for gene prediction with default parameters. EVidenceModeler [29] was used with a weight of 1:1:1 to combine the *ab initio* gene predictions of *Eimeria* spp.

The predicted genes were annotated using blast+ v2.7.1 searches against the core nucleotide database, and the results were integrated using Blast2GO [30]. Proteins containing signal peptides and transmembrane domains were predicted using SignalP v5.0 [31] and TMHMM v2.0 [32], respectively. PredGPI [33] was used to identify GPI-anchored proteins, while NetOGlyc-4.0 [34] was used to predict O-glycosylation sites. The online databases Kyoto Encyclopedia of Genes and Genomes (KEGG) (https://www.genome.jp/kegg/) and Pfam v36.0 (http://pfam.xfam.org/) [35] were used to annotate genes with catalytic functions and functional domains. Organellar genome sequences were not included in the protein function and pathway analyses. Metabolic pathway analysis was performed using the web server KEGG Automatic Annotation Server (KAAS) [36] with the bidirectional best hit setting and the eukaryotic model. MEME Suite v5.1.1 [37] was used to search for TA4 surface antigens and rabbit-specific *Eimeria* membrane proteins. All bioinformatic analyses were performed using the default settings, unless otherwise noted.

### Comparative genomic analyses

LTR transposons were predicted using LTRpred (https://github.com/HajkD/LTRpred/) and short tandem repeats (STRs) were predicted using RepeatMasker v4.1.1 (http://repeatmasker.org/). Homologous genes between *Eimeria* species were identified using OrthoMCL v2.0.9 and OrthoFinder v2.5.5 [38]. Venn diagrams showing shared and unique orthogroups among *Eimeria* spp. were generated using VennPainter [39]. Genome alignment was performed using Mauve v2.4.0 [40] with the default settings. An all-by-all blast+ v2.7.1 alignment was constructed to generate the appropriate input for MCScanX [41].

The amino acid sequences of single-copy orthologues among *Eimeria* spp. and other coccidia were concatenated for phylogenetic analysis using EasySpeciesTree.py (https://github.com/dongwei1220/EasySpeciesTree) with 1,000 bootstrap replicates. Invasion-related proteins identified from the Pfam domain search were aligned using muscle [42], with poorly aligned regions being trimmed using trimAl [43]. Phylogenetic trees were similarly constructed using Randomized Axelerated Maximum Likelihood (RAxML) [44] and Mega X [45], with 1,000 replicates for bootstrapping.

AlphaFold 3 [46] was used to predict the structure of MAR domains by homology modelling. The 3D structures of MARs were constructed using PyMOL v2.5.4 [47].

## Results

### Compact genome of *E. stiedae* compared to intestinal *Eimeria* species

**Fig. 1.**
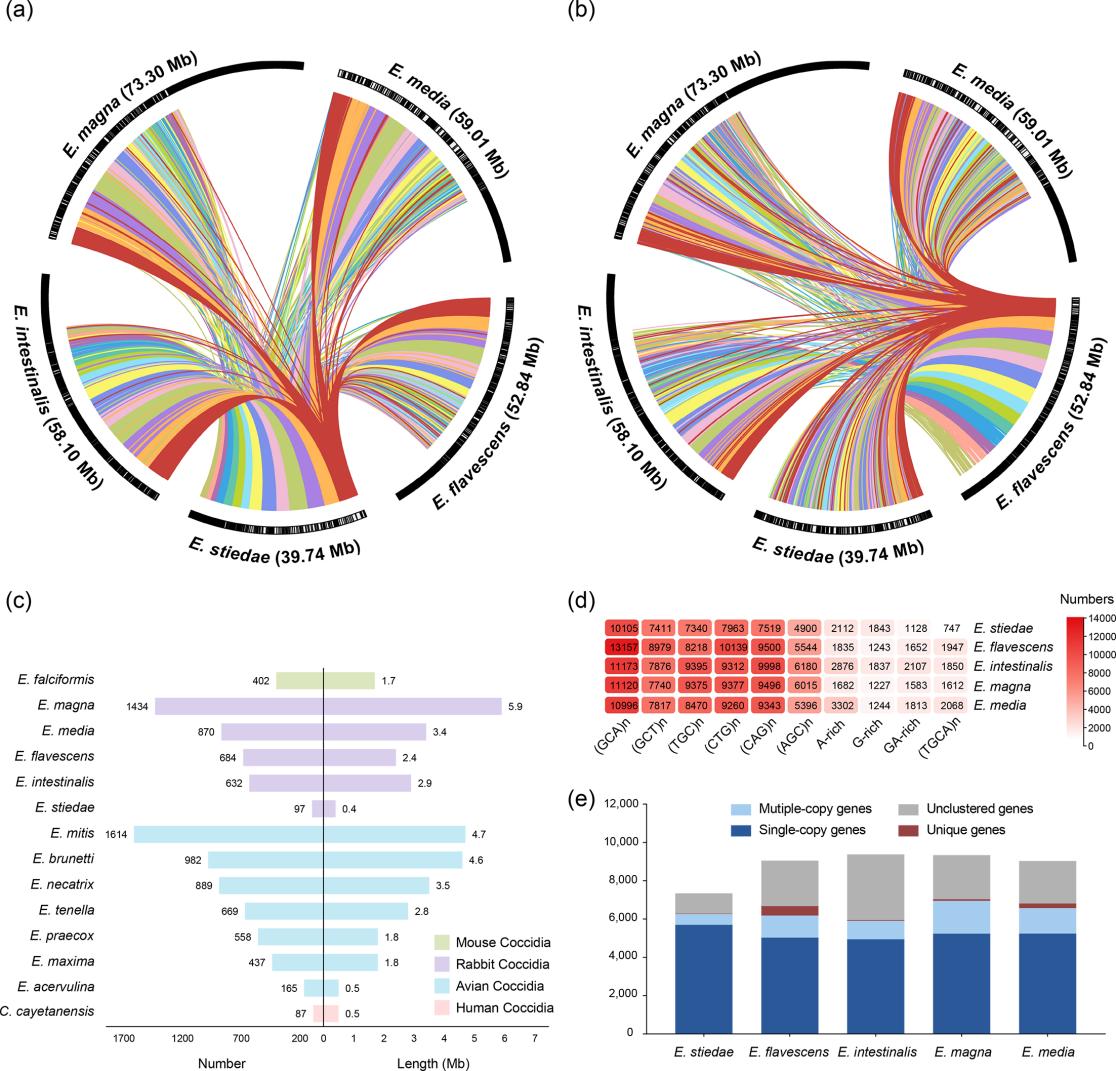
Genome features of five *Eimeria* species from rabbits. (**a**) Synteny among genomes of rabbit *Eimeria* spp. using *E. stiedae* as the reference. Collinear regions are connected by coloured lines. (**b**) Synteny among genomes of rabbit *Eimeria* spp. using *E. flavescens* as the reference. The black rectangular outlines indicate the boundaries between different contigs. (**c**) Differences in the number and length (Mb) of LTRs among *Eimeria* spp. and other common coccidia. (**d**) Distribution of the top ten STRs in rabbit *Eimeria* spp. (**e**) Distribution of different types of orthologues in rabbit *Eimeria* spp.

The rabbit *Eimeria* species examined in this study, including *E. stiedae*, *E. flavescens*, *E. intestinalis*, *E. magna* and *E. media*, were identified by oocyst morphology (Fig. S1, available in the online Supplementary Material). The genomes of these *Eimeria* species were sequenced using Illumina, yielding raw WGS data of 5.2 to 7.5 Gb for each. After *de novo* assembly of the genomes using a combination of CLC Genomics Workbench and SPAdes and removal of contaminants using blast, the species identity was further confirmed based on 18S rRNA and ITS-1 sequences (Table S1). The final assembly of the hepatic species *E. stiedae* was 39.7 Mb in length, consisting of 772 contigs with an N50 of 233,821 bp. This was shorter than the 52.8–73.3 Mb assemblies of the four intestinal *Eimeria* species, which have 2,270–5,791 contigs and N50 values of 90,771–188,909 bp (Table 1). BUSCO analysis of the data against the 446 core apicomplexan genes revealed that all 5 *Eimeria* genomes were >90% complete (Fig. S2). Good synteny was observed between *E. stiedae*, *E. intestinalis*, *E. magna* and *E. media* genomes (Fig. 1a), whereas lower collinearity was observed between these genomes and the *E. flavescens* genome (Fig. 1b). The assembled *E. stiedae* genome contained 6,893 protein-coding genes, compared to 7,672–8,450 in the other 4 *Eimeria* species. The percentage of coding regions in the *E. stiedae* genome was 37.6%, compared to 19.6–29.6% in the intestinal *Eimeria* species (Table S2). Furthermore, *E. stiedae* had shorter intergenic regions compared to the other species, while no significant difference was observed in intron length (Table S2).

**Table 1.** Statistics of genome assemblies of five rabbit *Eimeria* species

Category	Species	Total length (Mb)	No. of contigs	Largest contig	N50	N75
Raw assembly	*E. stiedae*	39.9	1,071	994,608	233,126	130,992
	*E. flavescens*	52.8	2,941	598,171	124,812	43,121
	*E. intestinalis*	58.4	2,693	457,970	90,133	42,407
	*E. magna*	73.5	6,076	623,563	91,184	29,182
	*E. media*	66.1	12,110	1,084,304	139,236	17,160
Draft genome*	*E. stiedae*	39.7	772	994,608	233,821	131,313
	*E. flavescens*	52.8	2,858	589,171	124,812	43,836
	*E. intestinalis*	58.1	2,270	457,970	90,771	43,583
	*E. magna*	73.3	5,791	623,563	91,590	29,700
	*E. media*	59.0	3,948	1,084,304	188,909	39,124

*The draft genome is the assembly after removing contigs of foreign DNA.

The difference in genome size between *Eimeria* spp. was partly due to differences in the number of repetitive sequences. The *E. stiedae* genome had only 97 LTRs with a total length of 0.4 Mb, whereas the genomes of 4 intestinal *Eimeria* species had 632–1,434 LTRs with a total length of 2.4–5.9 Mb (Fig. 1c). Pearson correlation analysis indicated that the more compact genome of *E. stiedae* was associated with a lower abundance and reduced length of LTRs, as well as shorter intergenic regions (Fig. S3). In contrast, the distribution of STRs among rabbit *Eimeria* spp. was similar (Fig. S4). ‘GCA’ was the most abundant STR (Fig. 1d). We further analysed the distribution of the STR ‘GCA’ and its variants (‘CAG’ and ‘AGC’) across the genomes. The results showed that these motifs were more frequently located in coding sequences than in intergenic regions (Fig. S5). In addition, *E. stiedae* had on average 728 fewer multi-copy genes and 585 more single-copy genes than the 4 intestinal *Eimeria* species, making the *E. stiedae* genome more compact (Fig. 1e). *E. stiedae* also had fewer genes encoding proteins with signal peptides, transmembrane domains and GPI anchors (Table S2).

### Evolutionary relationships and metabolic differences among *Eimeria* species

**Fig. 2.**
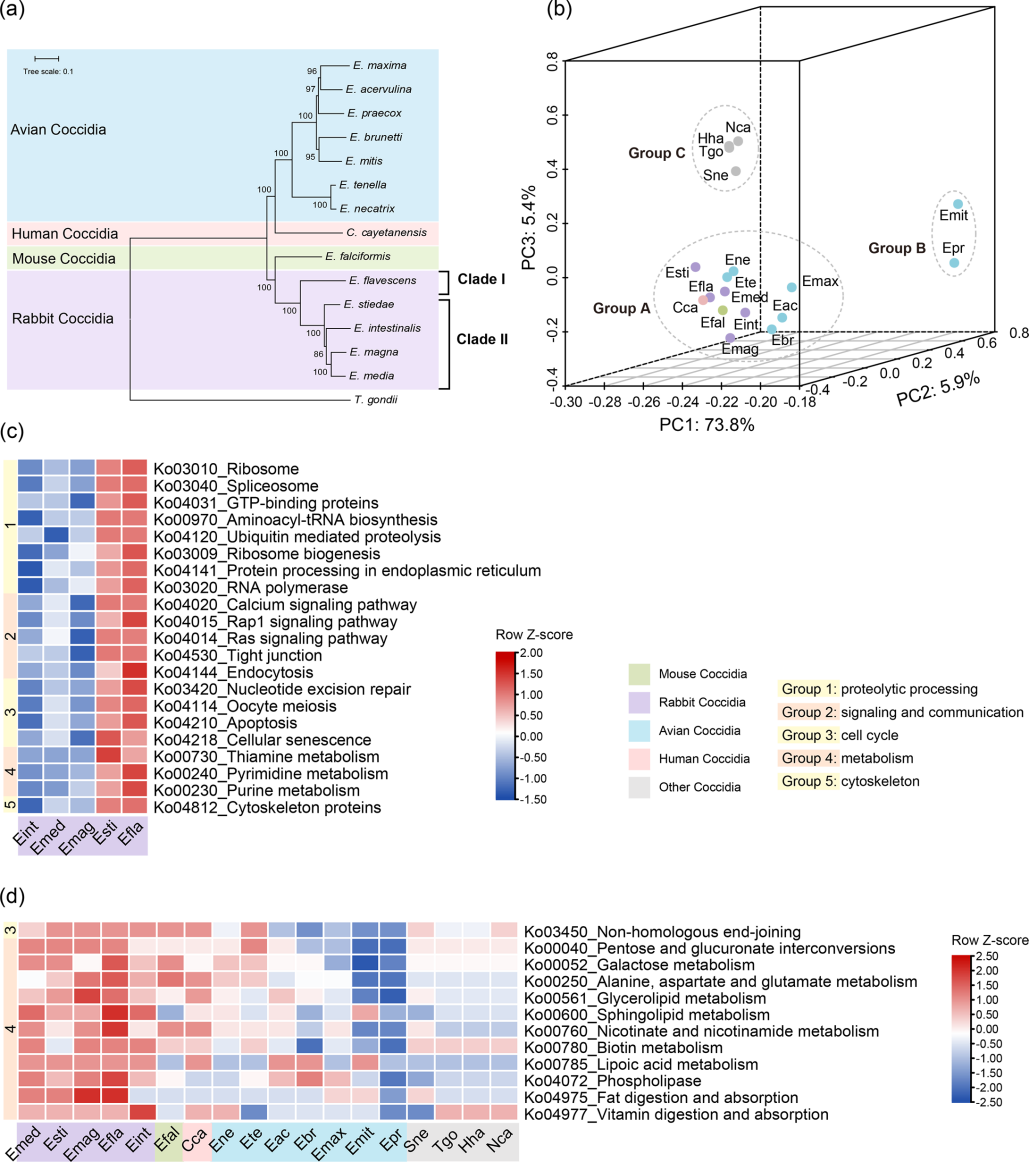
Differences in phylogenetic relationships and metabolic pathways among common coccidia. (**a**) Phylogenetic relationships of common coccidia based on maximum likelihood analysis of 106 single-copy orthologues of the genome. Bootstrap values (>80%) are shown at nodes. (**b**) Differences in metabolism among common coccidia based on principal component analysis of KEGG pathways. (**c**) Pathway completeness among rabbit *Eimeria*. Colour gradient indicates the row-scaled (Z-score normalization) of gene counts (blue, below mean; red, above mean). A higher number of pathway-associated genes suggest greater functional completeness. Functional categories: group 1, proteolytic processing; group 2, signalling and communication; group 3, cell cycle; group 4, metabolism; and group 5, cytoskeleton. (**d**) Comparison of pathway completeness between rabbit *Eimeria* and other coccidia.

Phylogenetic analysis of 106 single-copy orthologues of coccidia from different hosts (including 5 rabbit coccidia, 7 avian coccidia, *E. falciformis*, *Cyclospora cayetanensis* and *T. gondii*) revealed that the 5 rabbit *Eimeria* spp. were closely related to *E. falciformis* from mice. As expected, the five rabbit *Eimeria* species and the seven avian *Eimeria* species formed separate clusters, consistent with the host specificity of *Eimeria* spp. (Fig. 2a). In addition, *E. stiedae* was closely related to *E. intestinalis*, *E. megna* and *E. media*, while *E. flavescens* formed a separate branch. Phylogenetic analysis of the mitochondrial and apicoplast genomes revealed a similar relationship among rabbit *Eimeria* spp. (Fig. S6).

The results of principal component analysis showed that coccidia could be divided into three groups based on the completeness of KEGG pathways (Fig. 2b). Rabbit *Eimeria* had KEGG pathways similar to the majority of the coccidia and were clustered in group A, while two avian *Eimeria* and cyst-forming coccidia were clustered in groups B and C, respectively. However, some differences in metabolism were observed among rabbit *Eimeria* spp. For example, *E. stiedae* and *E. flavescens* possessed more genes in proteolytic processing, signalling and communication, cell cycle and metabolic and cytoskeleton pathways (Fig. 2c, Table S3). In addition, rabbit *Eimeria* spp. had more complete metabolic and DNA repair-related pathways than other coccidia (Fig. 2d, Table S4).

### Nature of genes unique to rabbit *Eimeria* species

**Fig. 3.**
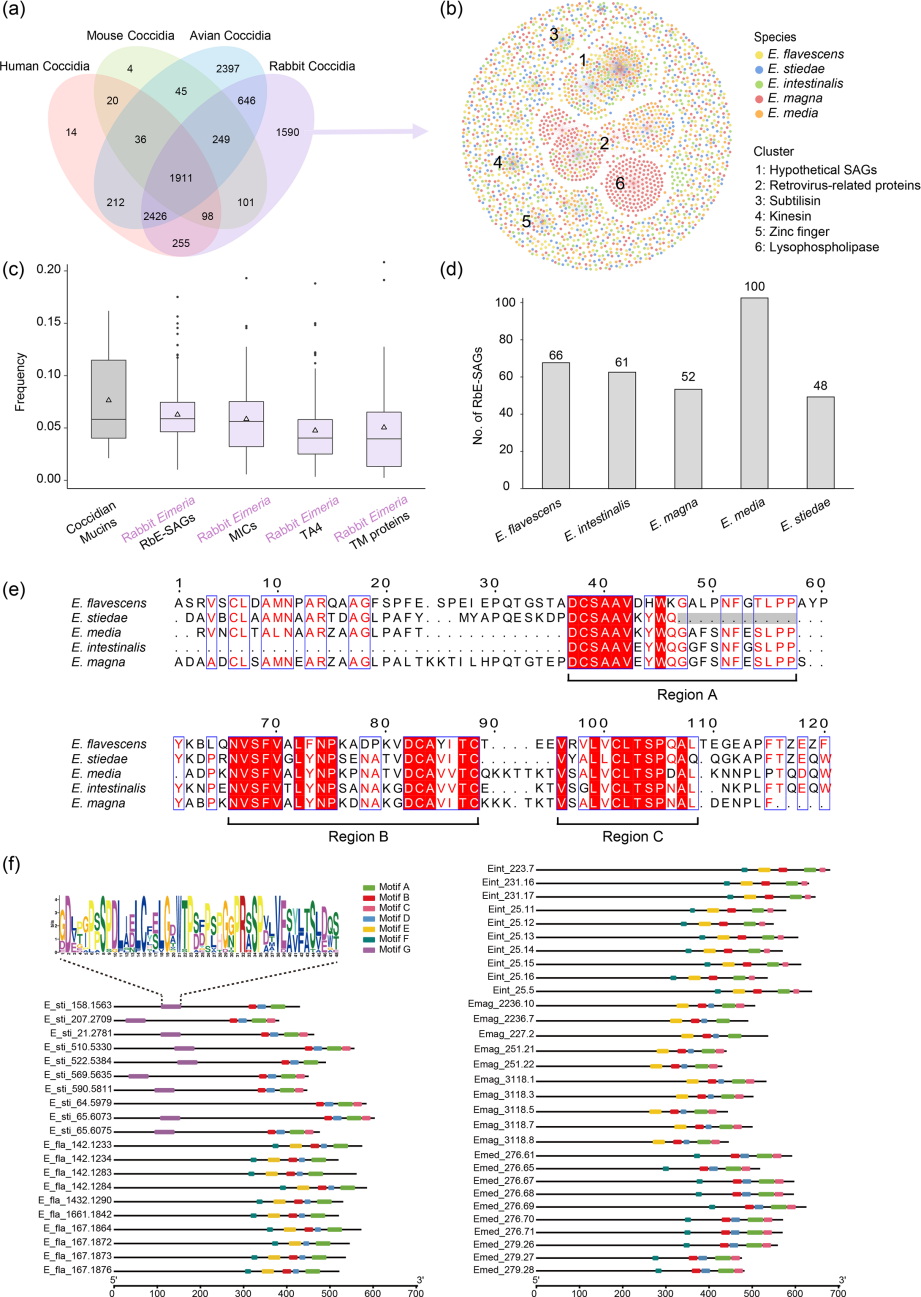
Species-specific orthologues and surface antigens in rabbit *Eimeria* spp. (**a**) Venn diagram showing shared and specific orthologues of *Eimeria* spp. There are 1,590 specific orthologous genes of rabbit *Eimeria*, including 214 single-copy genes. (**b**) Protein network based on the 1,590 orthologues of rabbit *Eimeria*. Each dot represents a protein. Cluster 1, hypothetical SAG proteins; cluster 2, retrovirus-related proteins, which are mostly seen in *E. magna*; cluster 3, subtilisin protein family; cluster 4, kinesin; cluster 5, zinc finger protein family. (**c**) Frequency of O-glycosylated sites in major groups of proteins of rabbit *Eimeria* spp. and other coccidia. (**d**) Number of hypothetical SAG proteins in rabbit *Eimeria* spp. (**e**) Alignment of high-frequency motifs in TA4 antigen sequences. Regions A, B and C represent the three conserved sequences. Red background indicates highly conserved regions; blue boxes represent regions with conservative substitutions. (**f**) Top five frequency motifs of all hypothetical SAG proteins of each rabbit *Eimeria* species. Motifs A–F are present in a wide range of species, while motif G is specific to *E. stiedae*. Only ten genes are displayed for each species.

There were 1,590 orthologous gene families unique to the 5 rabbit coccidia, including 214 single-copy genes (Fig. 3a). More than half of these unique genes were annotated as encoding hypothetical proteins (Tables S4 and S5). In network analysis of amino acid sequences, these unique orthologues of rabbit *Eimeria* spp. formed five major clusters, including hypothetical proteins (cluster 1), retrovirus-related proteins (cluster 2), subtilisins (cluster 3), kinesins (cluster 4), zinc finger proteins (cluster 5) and lysophospholipase (LPL) (cluster 6) (Fig. 3b). Zinc finger proteins, subtilisins and kinesins are involved in replication and egress of apicomplexans [48,51]. Therefore, these unique protein families may contribute to the adaptation of *Eimeria* spp. to rabbits. Cluster 6 comprises 166 expanded genes in *E. magna*. Among these, 105 genes encode hypothetical proteins, while 51 genes encode LPL. The remaining genes were annotated as retrovirus-related or transposon elements.

Approximately half of the hypothetical proteins (48.0–59.6%) in cluster 1 had transmembrane domains but only a small percentage (1.5–8.3%) had signal peptides (Table S5). In addition, members of this family were highly O-glycosylated (Fig. 3c), suggesting that these proteins were likely to be surface glycoproteins. These proteins were therefore considered to be novel SAGs (RbE-SAGs) unique to rabbit *Eimeria* spp. As expected, they differed in copy numbers between the five rabbit *Eimeria* species (Fig. 3d)

### Differences in surface proteins between hepatic and intestinal *Eimeria* species

In the functional domain analysis, the most diverse functional domains were related to surface antigens and adhesion (group 4) (Fig. S7). In particular, *E. stiedae* had only 17 proteins with the TA4 domain, while the intestinal *Eimeria* species had 24–49 (Fig. S7). The TA4 antigens of these five *Eimeria* species had three conserved regions (A, B and C). Among them, *E. stiedae* lacked the amino acid motif of GxxxNFxxLPP in region A (Fig. 3e).

Similarly, there were differences in the distribution of conserved sequence motifs within the RbE-SAGs unique to rabbit *Eimeria* spp. While motifs A to D were present in sequences from most rabbit *Eimeria* spp., motif G was present only in *E. stiedae* SAGs. Furthermore, motifs E and F, which were present in most intestinal species, such as *E. intestinalis* and *E. flavescens*, were absent in *E. stiedae* (Figs 3f, S8). This was further supported by differences in other orthologous genes between these two groups of coccidia. Among the 34 orthologous gene families of 148 protein-coding genes unique to intestinal *Eimeria* species, 2 orthologous gene families (OG39 and OG4528) of 19 genes encoded SAGs and sporozoite antigens with no other functional annotations (Tables S6 and S7).

### Sequence characteristics and evolutionary relationships of other invasion-associated proteins of *Eimeria* species

**Fig. 4.**
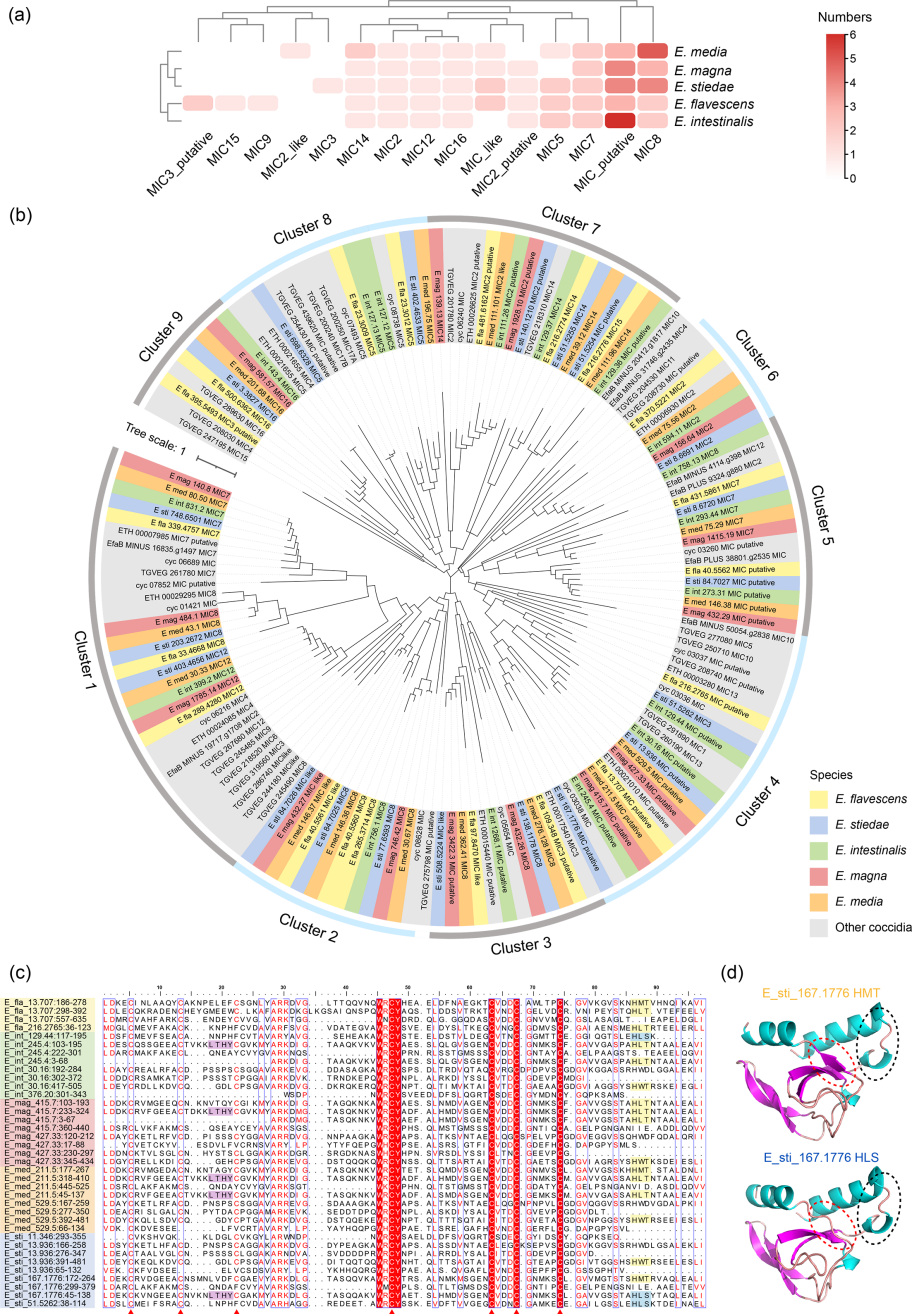
Features of MICs and MAR domains of rabbit *Eimeria* spp. (**a**) Copy numbers of MIC genes among rabbit *Eimeria* species. (**b**) Phylogenetic relationships of MICs among rabbit *Eimeria* spp. and other coccidia. (**c**) Sequence alignment of the conserved functional region within MAR domains from different *Eimeria* species. Conserved cysteine (Cys) residues are indicated by red triangles, conserved LxxY motifs are highlighted in purple and functionally active sialyl adhesive motifs (HxT) are highlighted in yellow, while inactive variants (HxS) are highlighted in blue (x indicates any amino acid residue). (**d**) 3D structure analysis of the MAR domain of *E. stiedae* with complete motifs. The red dotted line indicates the HxT/HxS motif, while the black dotted line indicates the LxxY motif with the *α*1-helix/loop extension.

The invasion-associated proteins of rabbit *Eimeria* spp. mainly include MICs, ROPs, RONs, GRAs and SAGs. Transcriptome analysis of sporulated and unsporulated *E. stiedae* oocysts revealed higher expression levels of invasion-related proteins in sporulated oocysts, particularly RbE-SAGs, MICs and ROPs (Fig. S9a). Members within the RbE-SAG family displayed divergent expression patterns between sporulated and unsporulated oocysts; while the majority of genes were significantly upregulated, some genes were downregulated or showed no significant change (Fig. S9b). Of the 15 identified MICs, MIC7 and MIC8 and 1 putative MIC had multiple copies, while the others were absent in some *Eimeria* spp. (Fig. 4a). Based on the phylogenetic relationships, the MICs of rabbit *Eimeria* spp. were grouped into nine clusters (Fig. 4b). Domain analysis revealed that the MICs in cluster 4 had an MAR domain that binds sialic acid (Sia). Sequence alignment revealed that the MAR domains of rabbit *Eimeria* spp. contain seven conserved cysteine (Cys) residues (Fig. 4c). 3D structural analysis showed that the LxxY motif displayed a characteristic *α*1-helix/loop structure (Fig. 4d). These features were highly conserved among *Eimeria* MAR domains, identifying them as type I MAR domains. Most MAR domains of MICs in cluster 4 were found to have HxT/HxS motifs (Fig. 4c). The MAR domains of hepatic *Eimeria* had only two HxT sequences with Sia-binding activity, whereas intestinal *Eimeria* had three or more.

**Fig. 5.**
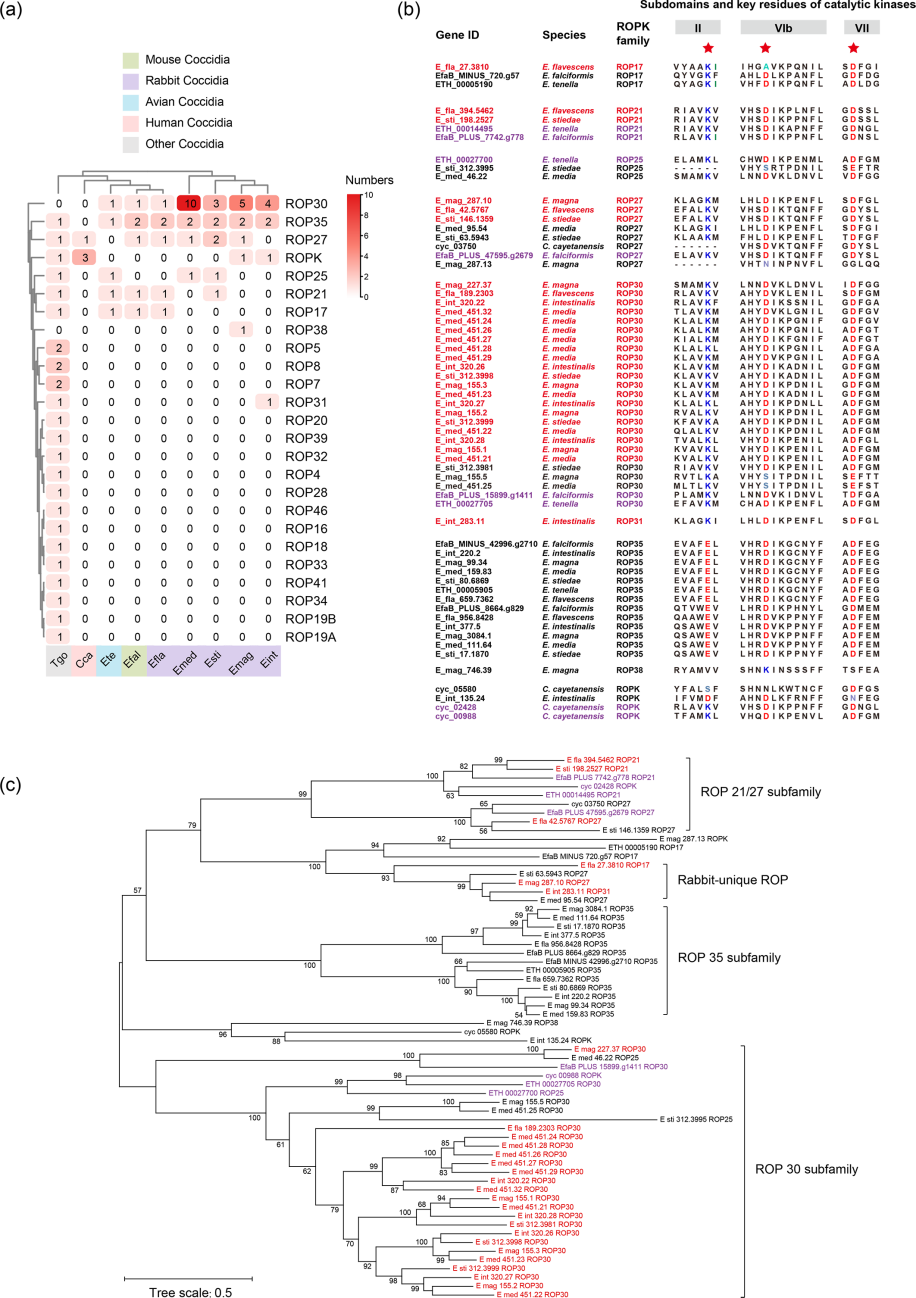
Sequence features and phylogenetic relationships of ROPs of rabbit *Eimeria* spp. (**a**) Copy numbers of ROP genes among common coccidia. (**b**) Alignment of the active sites of ROPs among common coccidia. Genes with putative ROPK activity are highlighted in red for rabbit *Eimeria* spp. and purple for other *Eimeria* spp. On the right are three subunits (II, VIb and VII) of eukaryotic kinases with catalytic functions. Among them, lysine (K) is the key amino acid of subunit II, and aspartic acid (D) is the key amino acid site of both VIb and VII. (**c**) Phylogenetic relationships of ROPs among rabbit *Eimeria* spp. and other coccidia. Red font represents the activated rhoptry kinase of rabbit *Eimeria* spp., and purple font represents the activated rhoptry kinase of other *Eimeria* spp.

The ROPs of five rabbit *Eimeria* spp. mainly belonged to the ROP30, ROP35, ROP27, ROP25 and ROP21 subfamilies (Fig. 5a). Among these, ROP30, ROP27 and ROP21, which possess kinase catalytic function, retained the conserved residues ‘K-D-D’ at three key sites. In contrast, ROP35 had an amino acid substitution (K to E) at one critical residue (Fig. 5b). Since the ROP30 subfamily had the most members, ROP30 proteins were likely to be the primary functional ROP kinases in rabbit *Eimeria* spp. Of the *Eimeria* spp. analysed, *E. media*, *E. magna*, *E. intestinalis*, *E. stiedae* and *E. flavescens* had 10, 5, 4, 3 and 1 ROP30 genes, respectively. These results suggest variations in the copy number of genes encoding active rhoptry kinases (ROPKs) among rabbit *Eimeria* spp. In the phylogenetic analysis, the ROPKs of rabbit *Eimeria* spp. formed four clusters, including ROP21/27, ROP35, ROP30 and rabbit-unique ROP (Fig. 5c). There were no significant differences in the copy number of RONs between hepatic and intestinal *Eimeria* species. Among the ten RON genes identified, RON3, RON4 and RON5 had multiple copies (Fig. S10a). These genes formed seven clusters in the phylogenetic analysis of amino acid sequences, with no species-specific RONs (Fig. S10b).

Gene annotation revealed that the GRAs of rabbit *Eimeria* included GRA9, GRA10, GRA11 and GRA12. They formed four clusters in the phylogenetic analysis of the sequences. *E. stiedae* lacked one copy of GRA11 compared with the four intestinal *Eimeria* species (Fig. S11a). Most GRAs of the four intestinal *Eimeria* species contained signal peptides and regions of low complexity (Fig. S11b). In contrast, GRA10, GRA11 and GRA12 of *E. stiedae* lacked signal peptides, indicating fewer secretory GRAs in the hepatic species.

Of the 13 major groups of proteins with known functional domains in common coccidia, those related to RNA synthesis and translation (group 2) were the most abundant and relatively conserved (Fig. S7). Kinases (group 1) were also abundant, but most of the difference in numbers was between *Toxoplasma* and other coccidia. There were only minor differences in copy numbers between the five rabbit *Eimeria* spp. However, *E. stiedae* had more proteins with calcium-binding EF-hand_7 motif (group 10) than the intestinal *Eimeria* species did (26 in *E. stiedae* compared to 13 to 19 in others, Fig. S7), suggesting that there may be some differences in calcium regulation of the secretion of invasion-related proteins between these two groups of parasites.

## Discussion

In this study, we have sequenced the genomes of five rabbit *Eimeria* spp. and discovered a compact genome of *E. stiedae* compared to the four intestinal *Eimeria* species. Protein function and pathway analyses suggest that the metabolic pathways of *E. stiedae* are similar to those of *E. flavescens*. Comparative genomic analyses reveal that *E. stiedae* possesses a novel class of surface proteins and fewer TA4 and other invasion-related proteins. These observations may be important for the understanding of the determinants of coccidial tissue tropism.

*E. stiedae* has a smaller genome and more single-copy genes than the intestinal *Eimeria* species. Consequently, its gene density is much higher than that of the four intestinal *Eimeria* species and other common coccidia, such as avian *Eimeria* spp., *C. cayetanensis* and *T. gondii*. Significant differences in genome size and gene content have also been reported among the seven chicken *Eimeria* spp. [52]. The smaller genome size of *E. stiedae* may be due to its fewer LTRs compared to the intestinal *Eimeria* species. This is consistent with the observation of a positive correlation between LTR retrotransposon content and genome size in many eukaryotic organisms [53,54]. The number of ‘GA-rich’ and ‘TGCA’ repeats in *E. stiedae* is also lower than in the four intestinal *Eimeria* species, though the distribution of other STRs is similar. These findings suggest that non-LTR repeats have a minimal impact on the genome size of *Eimeria* spp.

Despite exhibiting tissue tropism, rabbit *Eimeria* species do not form tissue-specific groups in phylogenetic analyses. A previous phylogenetic analysis of the 18S rRNA gene revealed that the 11 rabbit *Eimeria* species clustered into 2 major groups based on the presence or absence of the oocyst residuum [55]. Similarly, in the present analysis and in a previous phylogenetic analysis of single-copy orthologues [56], *Eimeria* species form host-associated clusters. In addition, *E. stiedae*, *E. intestinalis*, *E. magna* and *E. media* with an oocyst residuum cluster together, while *E. flavescens* without an oocyst residuum is placed on a separate branch. This clear morphological and phylogenetic distinction suggests that tissue tropism alone cannot explain the evolutionary relationships among these parasites. Rather, it supports the idea that other genetic and biological differences contribute to the diversification of rabbit *Eimeria* species.

The results of this study suggest that differences in metabolism are unlikely to significantly contribute to tissue tropism. The metabolism of the five rabbit *Eimeria* species is similar to each other’s and to that of some other coccidia (group A). This is consistent with previous observations of metabolic conservation between *T. gondii* and *N. caninum*, despite their very different tissue tropisms [57]. However, subtle differences exist between coccidia. For example, rabbit coccidia have a more complete energy metabolism than other coccidia, including phospholipase, vitamin and other metabolic pathways. In addition, *E. stiedae* and *E. flavescens* have more complete thiamine and nucleotide metabolism compared to other rabbit *Eimeria* spp. Thiamine, a critical precursor to thiamine pyrophosphate, is primarily obtained through scavenging and plays a role in amino acid metabolism in apicomplexans [58]. Coccidia are known to lack *de novo* purine synthesis and rely on the salvage pathway for nucleotide metabolism [59].

Interestingly, because bile has been shown to inhibit oocyst sporulation and sporozoite activity in coccidia [60], it is possible that *E. stiedae* has evolved unique mechanisms to tolerate or exploit bile-rich conditions. Data from this study revealed that the species-specific gene families in *E. stiedae* are predominantly composed of hypothetical proteins and retrotransposon-like proteins (Table S4). We hypothesize that these transposon-related elements may facilitate adaptation. Supporting this notion, ablation of the Tf2 retrotransposon in fission yeast was shown to impair fitness, underscoring the potential role of transposable elements in environmental adaptation [61]. Nevertheless, whether a similar, direct sensory mechanism exists in *E. stiedae* remains an open question for future research.

In addition, protein network analysis indicated that *E. magna* has an expanded gene family containing several genes encoding LPL, suggesting a potential functional involvement in lipid metabolic reprogramming and host immune response modulation. Although the biological significance of the numerous unannotated genes within this family remains unclear, the marked enrichment of LPL points to an association with lipid metabolic processes. The core function of PfLPL3 in *Plasmodium falciparum* is to break down scavenged host lipids into fatty acids, which are then used for the synthesis of neutral lipids [62]. These neutral lipids are subsequently mobilized in a timely manner to support schizogony and merozoite formation. Moreover, previous studies have demonstrated that recombinant EmLPL (rEmLPL) confers effective immune protection against *E. maxima* infection [63]. Whether this genetic expansion is directly linked to distinctive biological features of *E. magna* requires further functional investigation.

The reduction in the number of the TA4 surface antigens and the deletion of conserved amino acids in these proteins may make *E. stiedae* less infectious to intestinal epithelial cells than to biliary epithelial cells. The TA4 surface antigens belong to the SAG family, containing an N-terminal signal peptide and a C-terminal GPI [64]. SAG has been implicated in host cell invasion and tissue tropism in multiple studies [5,65, 66]. The SAGs of avian *Eimeria* can be divided into three subfamilies: SAGA, SAGB and SAGC, with SAGA being the core surface antigen of *Eimeria* [52]. However, the TA4 surface antigens of the five rabbit *Eimeria* species do not fit neatly into these three subfamilies and only show evolutionary similarities to some SAGA members. A comparison of the sequence motifs within the TA4 antigens among the rabbit *Eimeria* species shows that the hepatic *E. stiedae* lacks a conserved amino acid fragment of the sequence GxxxNFxxLPP, which is located within the functional domain of the SAGs. Thus, it is hypothesized that *Eimeria* species infecting different hosts and tissues have specific SAGs. In addition, *E. stiedae* has fewer membrane proteins than the four intestinal *Eimeria* species, which may contribute to its hepatic tropism. Notably, *E. stiedae* has significantly fewer proteins with the GPI anchor, which is a functional structure of some surface antigens in *Eimeria* spp. [52]. Consequently, *E. stiedae* has fewer surface proteins than in the four intestinal *Eimeria* species. Furthermore, an *E. stiedae*-specific motif was discovered among the new family of SAGs (RbE-SAGs) of rabbit *Eimeria spp*. These differences in conserved motifs in TA4 antigens and RbE-SAGs may contribute to the tissue tropism of hepatic and intestinal *Eimeria* species. Whether this motif is associated with tissue tropism in coccidia remains to be determined experimentally.

The expression profiles of invasion-related proteins in sporozoites may contribute to the adaptability and host specificity of *Eimeria* spp. during the infection stage. The MAR domains of *E. stiedae* contain fewer HxT motifs with salic acid (Sia)-binding activity, which may lead to differences in tissue tropism. MICs of coccidia possess HxT/HxS motifs in their MAR domains that exhibit sialyl adhesive activity [14]. In *E. tenella*, only the HxT and VxT motifs of the MAR domain can bind to Sia, while the HxS and YxE variants cannot [67]. TgMIC13 binds strongly to *α*2-9-linked disialyl on embryonic cells, whereas NcMIC1 prefers 4-O-acetylated Sia on the intestinal epithelium [68]. These findings suggest that MAR’s preference for different modified Sia may influence tissue tropism. In avian coccidia, MAR3 of EaMIC3, MAR2 of EmMIC3 and MAR4 of EmiMIC3 have been identified as determinants of tissue tropism [15]. In this study, we found that *E. stiedae* MAR domains have fewer HxT motifs. This suggests that *E. stiedae* MICs may bind to Sia less strongly than MICs of intestinal *Eimeria* species do. Further investigation of the Sia-binding ability of *E. stiedae* MAR domains is necessary to identify the specific MAR domains that influence tissue tropism.

*E. stiedae* is likely less adapted to the intestinal environment than other *Eimeria* species in rabbits [3,4]. In addition to having a more compact genome, it has fewer ROPKs than the four intestinal *Eimeria* species. ROPs are classified as ROPKs or pseudokinases based on the presence of kinase activity in the former and the absence of it in the latter [69]. Interestingly, although pseudokinases lack kinase activity, a previous study suggested that the pseudokinase ROP5 in *T. gondii* inhibits host immune responses in concert with the kinases ROP17 and ROP18 by forming a complex with them [70,71]. However, the ROP25 pseudokinase identified in *E. stiedae* is evolutionarily distant from its relatives in other *Eimeria* species. The lack of significant differences in the number and types of RONs among rabbit *Eimeria* species may be related to their conserved functions in host cell invasion. RONs are released at the pre-invasion stage and are involved in gliding and MJ formation, which are critical for establishing coccidial infection.

Other proteins are likely to also contribute to the biological differences among rabbit *Eimeria* species. For instance, *E. stiedae* lacks a copy of GRA11 and has lost the signal peptide and low-complexity regions of GRA12. In *T. gondii* and related cyst-forming coccidia, GRAs are translocated to the PVM or exported into host cells to regulate immune responses [72]. For instance, *T. gondii* GRA7, GRA14 and GRA15 stimulate Th1 responses in infected mice by activating the NF*κ*B signalling pathway, thereby reducing parasite virulence [73]. In contrast, targeted deletion of GRA 9 in *T. gondii* blocks cyst formation and reduces parasite virulence [74]. Thus, GRAs likely play distinct roles in coccidial growth and virulence. Further studies are needed to investigate whether the absence of specific GRAs and functional GRA domains affects the virulence or tissue tropism of *E. stiedae*.

In conclusion, this study has revealed a compact genome of *E. stiedae* and identifies genomic features potentially associated with its distinct tissue tropism. Specifically, compared to other intestinal *Eimeria* species, *E. stiedae* has a unique motif in the newly discovered family of SAGs (RbE-SAGs) and fewer functional domains of invasion-related proteins. These findings provide valuable insights into differences that may be associated with the invasion and tissue tropism mechanisms of *Eimeria* species. Future studies focusing on the molecular and cellular functions of these proteins will be essential to elucidate the precise mechanisms underlying *Eimeria* host adaptation and invasion pathways.

## Supplementary material

10.1099/mgen.0.001576 Uncited Supplementary Material 1.

10.1099/mgen.0.001576 Uncited Supplementary Material 2.

## Abbreviations

GRA: dense granule proteins
KEGG: Kyoto Encyclopedia of Genes and Genomes
LPL: lysophospholipase
MAR: microneme adhesive repeat
MIC: micronemal proteins
MJ: moving junction
NCBI: National Center for Biotechnology Information
PV: parasitophorous vacuole
PVM: parasitophorous vacuole membrane
RON: rhoptry neck
ROP: rhoptry bulb
ROPK: rhoptry kinases
SAG: surface antigens
Sia: sialic acid
STR: short tandem repeats
WGS: whole-genome sequence
