# Supplementary material for: Comparative genomic analysis of five Eimeria spp. in rabbits provides insights into coccidian tissue tropism
Source: Microb Genom. 2025 Nov 27;11(11):001576. doi: 10.1099/mgen.0.001576 (PMC12659853; doi:10.1099/mgen.0.001576)

**Supplementary Information**

# Comparative genomic analysis of five *Eimeria* spp. in rabbits provides insights into coccidian tissue tropism

Tianyi Hou[1], Dongle Su[1], Xinran Wang[1], Yanhua Xu[1], Junhong Lu[1], Qi Wang[1], Tianpeng Wang[1], Rui Xu[1], Yaqiong Guo[1], Na Li[1], Xun Suo[2], Yaoyu Feng[1]* and Lihua Xiao[1]*

[1] State Key Laboratory for Animal Disease Control and Prevention, Center for Emerging and Zoonotic Diseases, College of Veterinary Medicine, South China Agricultural University, Guangzhou 510642, China.

[2] College of Veterinary Medicine, China Agricultural University, Beijing 100083, China.

*__Correspondence:__ Yaoyu Feng, yyfeng@scau.edu.cn; Lihua Xiao, lxiao1961@gmail.com

**Supplementary Figure Legends:**

**Fig. S1.** Morphology of oocysts of five *Eimeria* species of rabbit. The bar represents 50 μm for *E. media* and 20 μm for others. Oocysts of *E. stiedae* are ellipsoidal, those of *E. magna* are ovoid, those of *E. intestinalis* are piriform, and those of *E. flavescens* and *E. media* are ovoid to ellipsoidal.

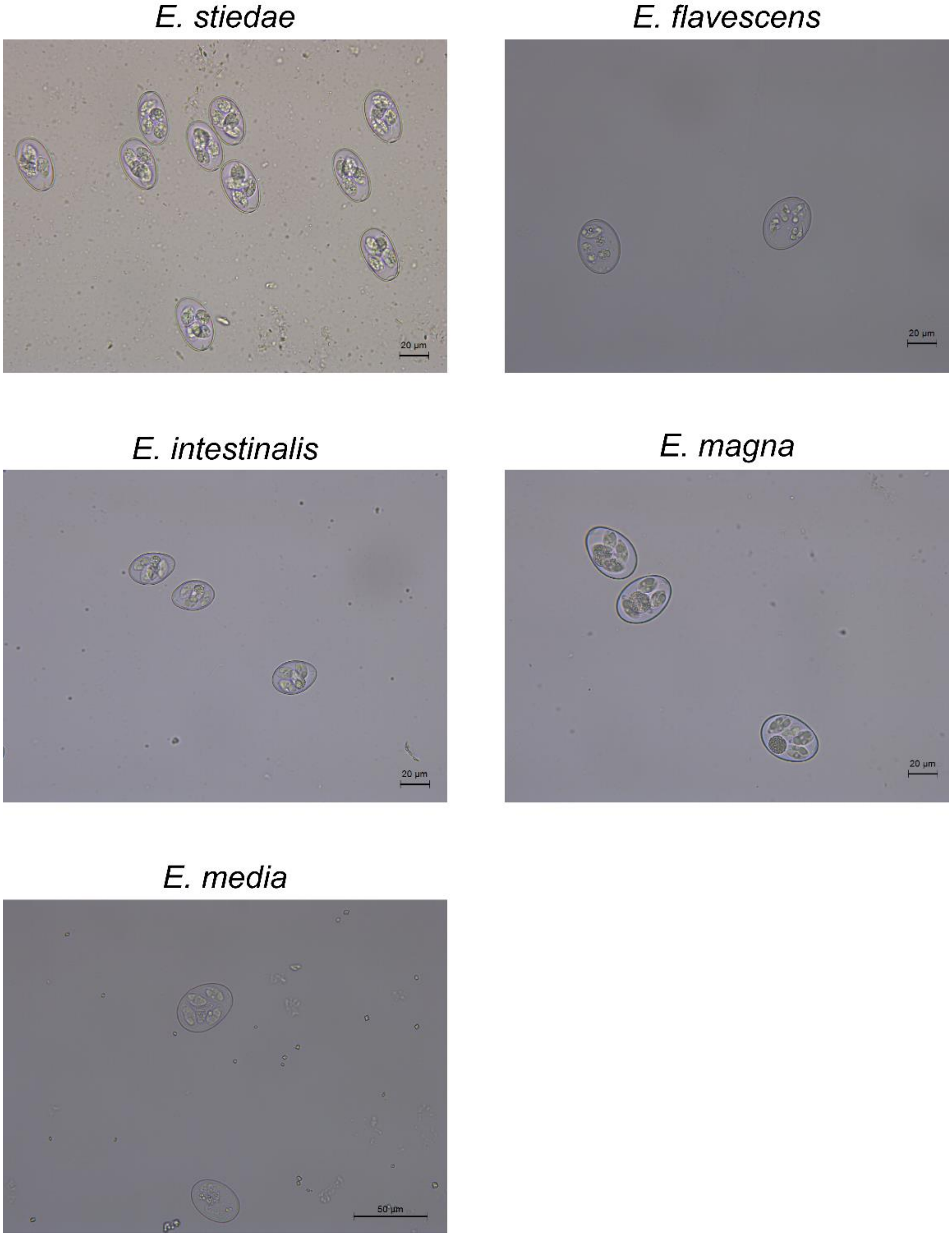

**Fig. S2.** Genomic integrity of *Eimeria* spp. based on 446 core genes of Apicomplexa. Complete and single-copy genes are indicated in light blue; complete and duplicated genes are indicated in dark blue. Fragmented genes are indicated in yellow, and missing genes are indicated in red. Higher complete single-copy BUSCO scores indicate better genomic integrity.

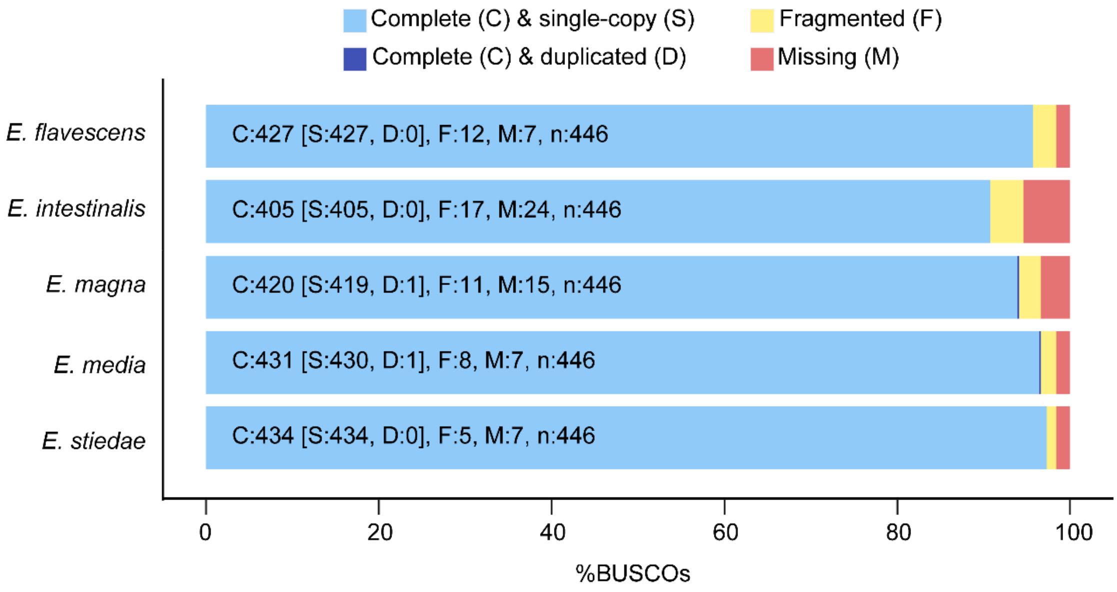

**Fig. S3.** Correlation between genome size and genomic features in *Eimeria* species as indicated by the Pearson correlation coefficients (r) and *p*-values of (a) total LTR length, (b) LTR counts, (c) average intergenic region length, and (d) average intron length. Significant positive correlations are observed in (a-c), while (d) shows no significant correlation.

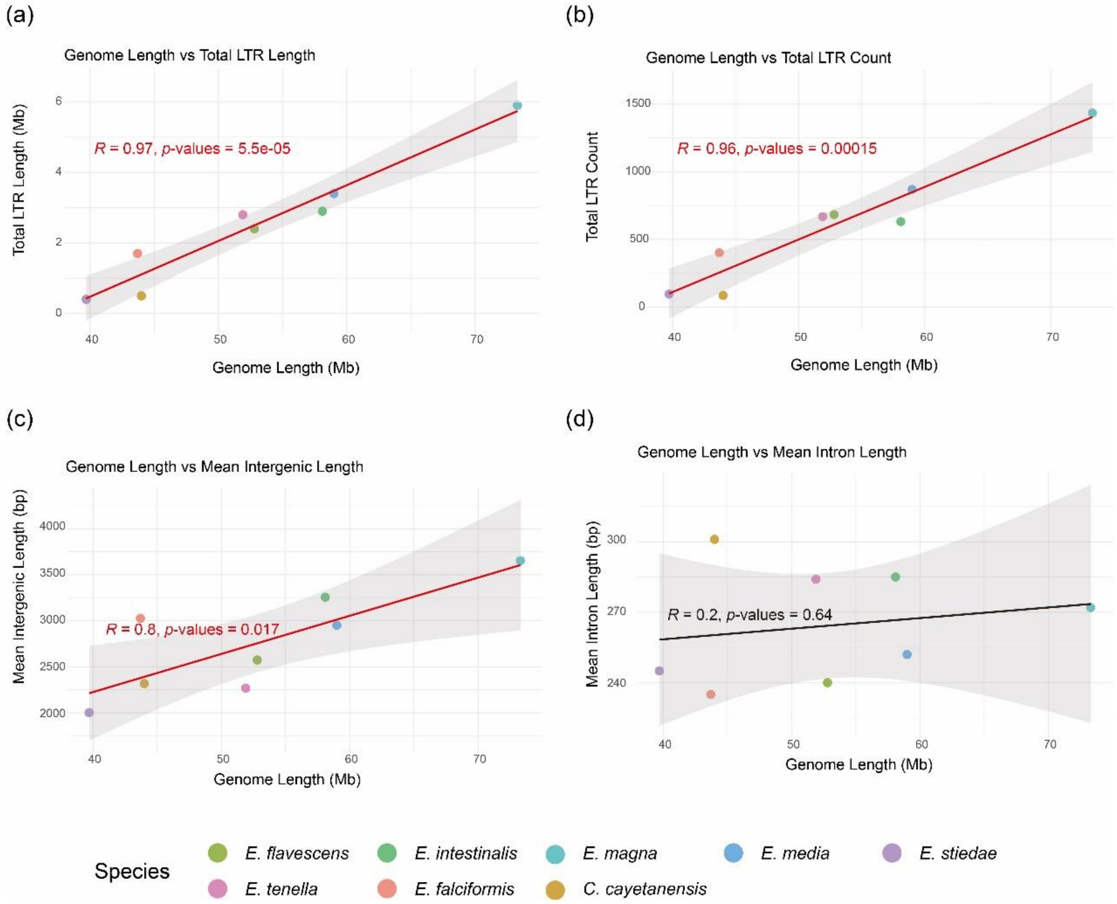

**Fig. S4.** Length of 10 top STRs in rabbit *Eimeria* spp. The x-axis indicates the type of STRs, and the y-axis shows the length of the STRs.

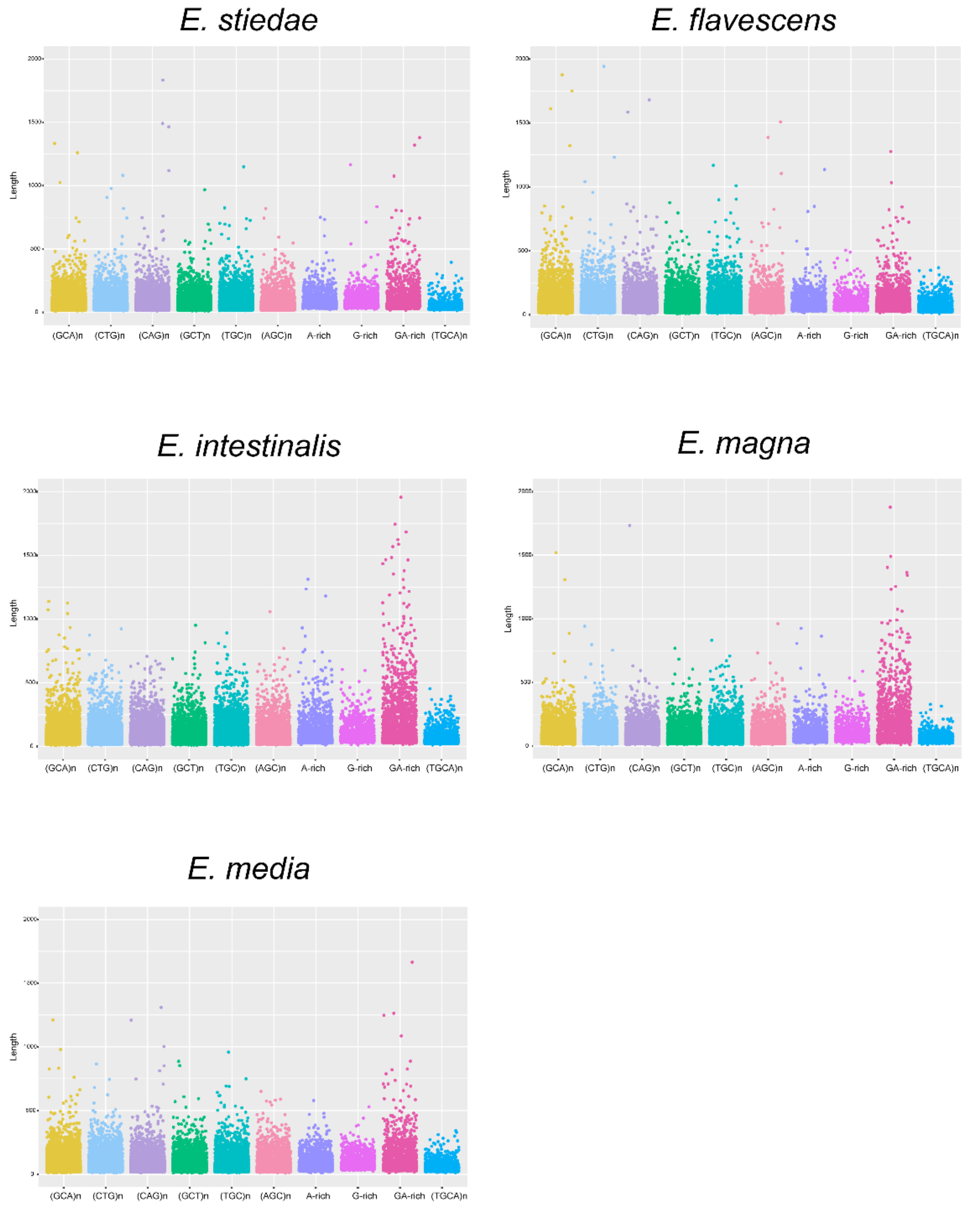

**Fig. S5.** Genomic distribution of some STRs in rabbit *Eimeria* spp. The pie charts illustrate the percentage distribution of "GCA", "CAG", and "AGC" across different genomic regions.

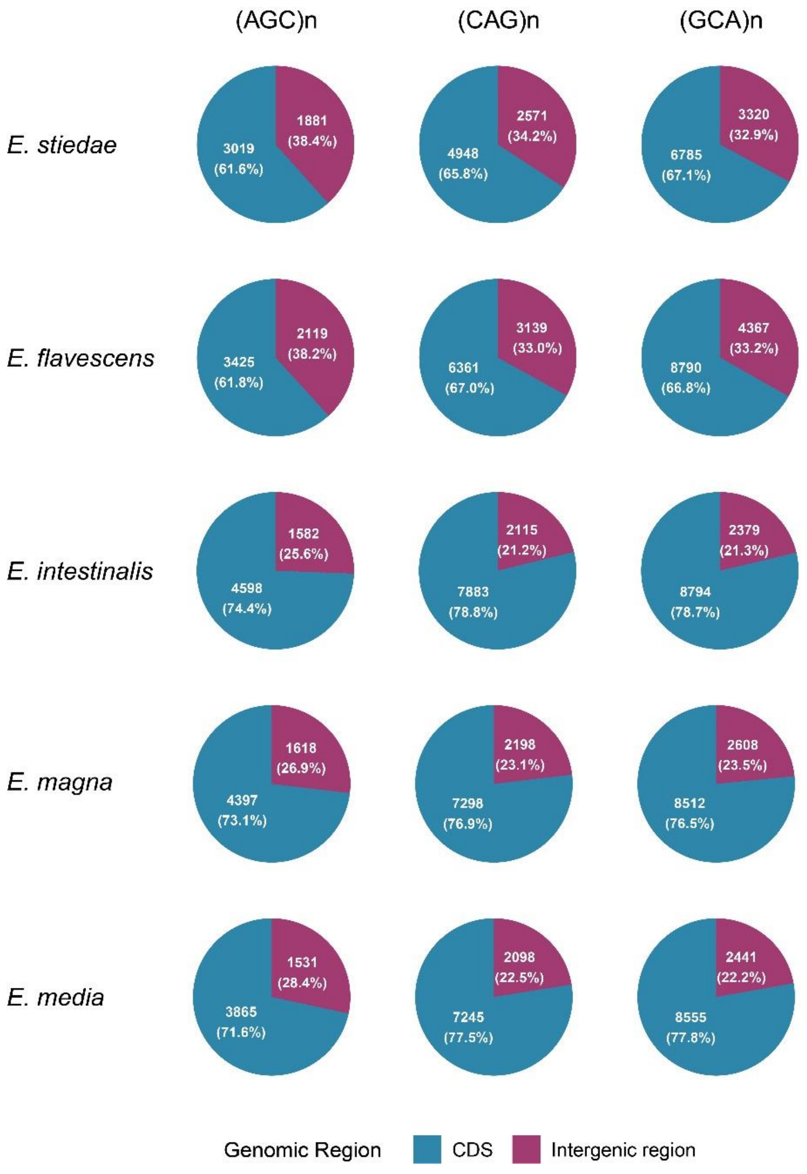

**Fig. S6.** Phylogenetic relationships of mitochondrial and apicoplast genomes. (a) Maximum likelihood tree of mitochondrial genomes and (b) apicoplast genomes. The bootstrap values (>50%) from 1,000 replicate analyses are shown at nodes. The mitochondrial and apicoplast genomes assembled in this study are highlighted in bold.

(a)

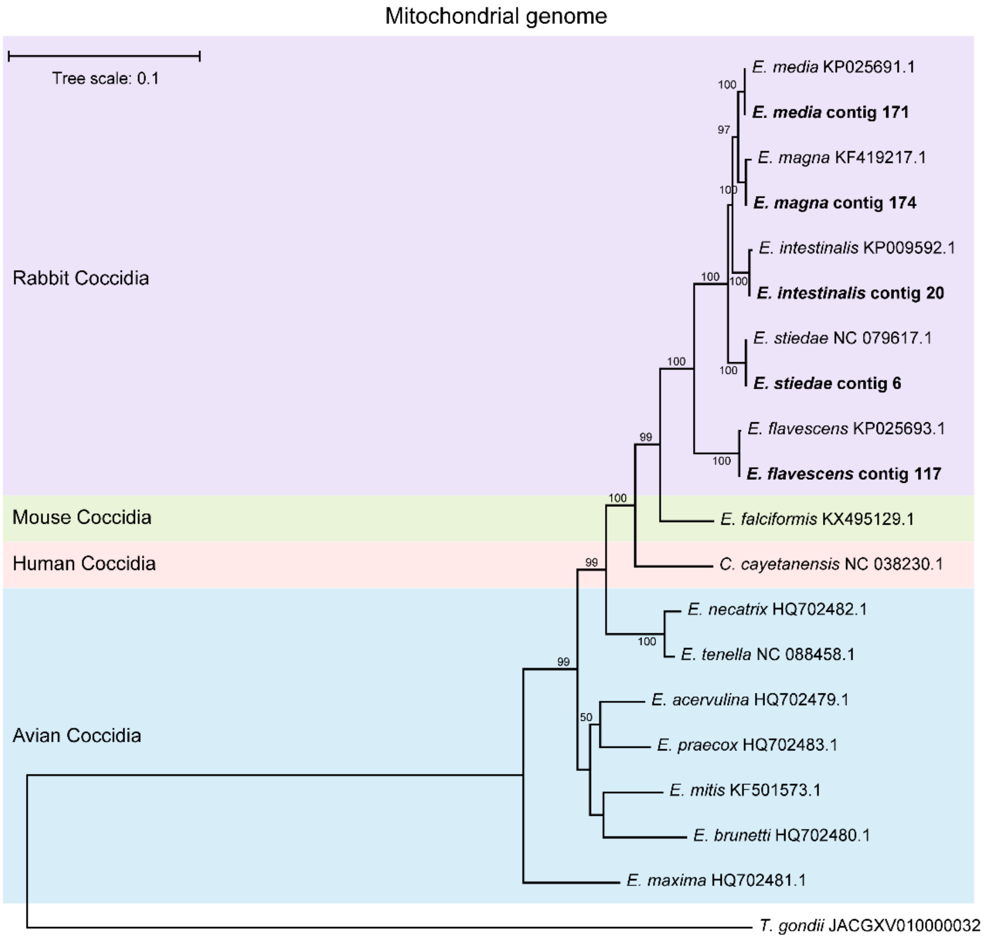


(b)

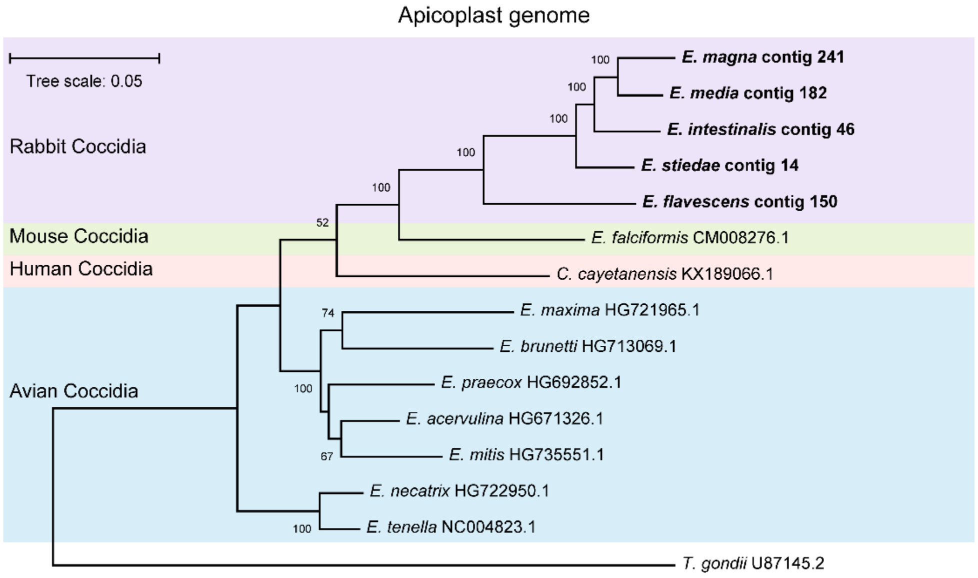

**Fig. S7.** Distribution of major Pfam domains in common coccidia based on the functions of the domains. Group 1: kinases; Group 2: RNA synthesis and translation; Group 3: membrane regulation; Group 4: surface antigens and adhesion factors; Group 5: peptidase and protease; Group 6: signaling pathways; Group 7: ubiquitination; Group 8: protein biosynthesis; Group 9: molecular chaperone; Group 10: regulation of intracellular calcium level; Group 11: driver protein; Group 12: metal-dependent protein phosphatase; Group 13: regulatory factors of chromosome aggregation.

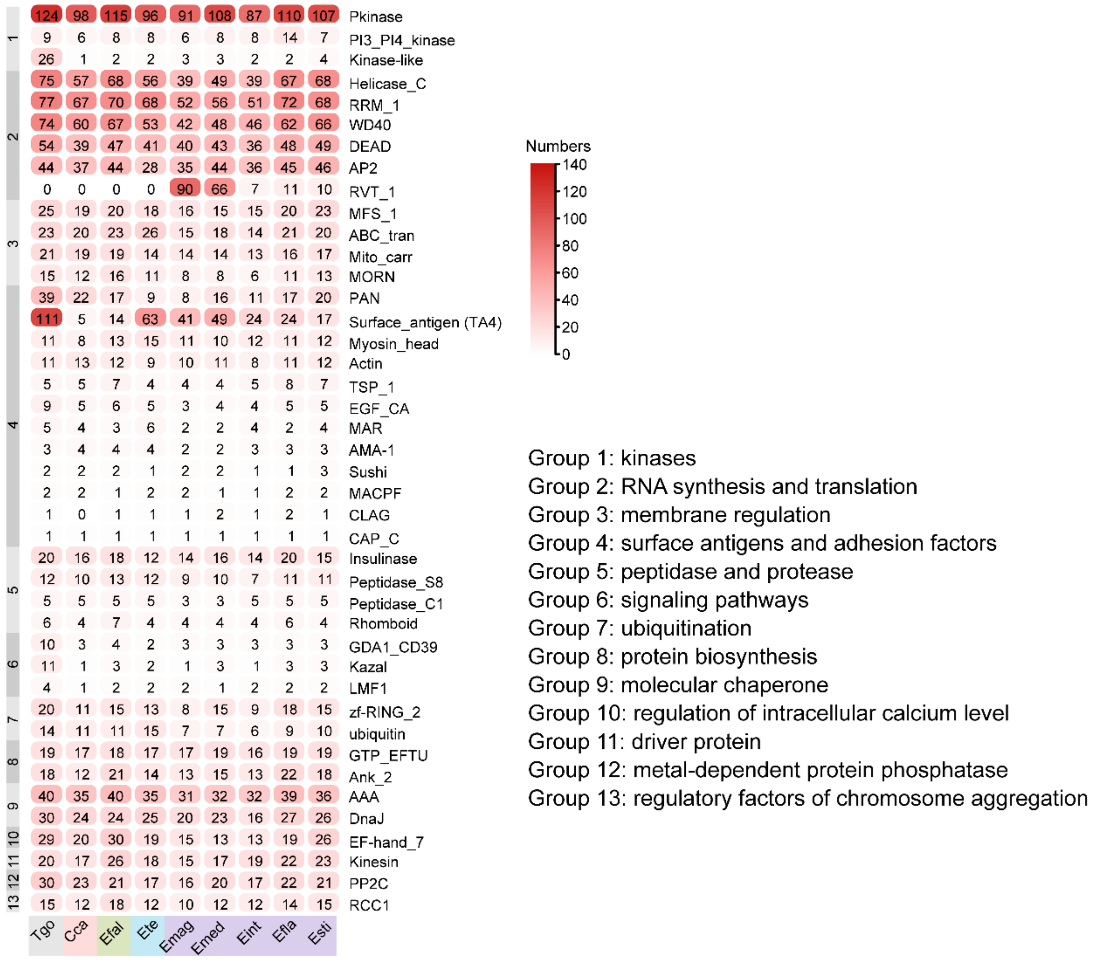

**Fig. S8.** Conserved motifs of hypothetical SAG proteins in each rabbit *Eimeria* species. (a) The five top motifs in hypothetical SAG proteins from each species. *E. stiedae* has a species-specific motif (Motif G), while motif E and F are more conserved in intestinal *Eimeria* spp. (b) Presence or absence of these motifs across rabbit *Eimeria* species.

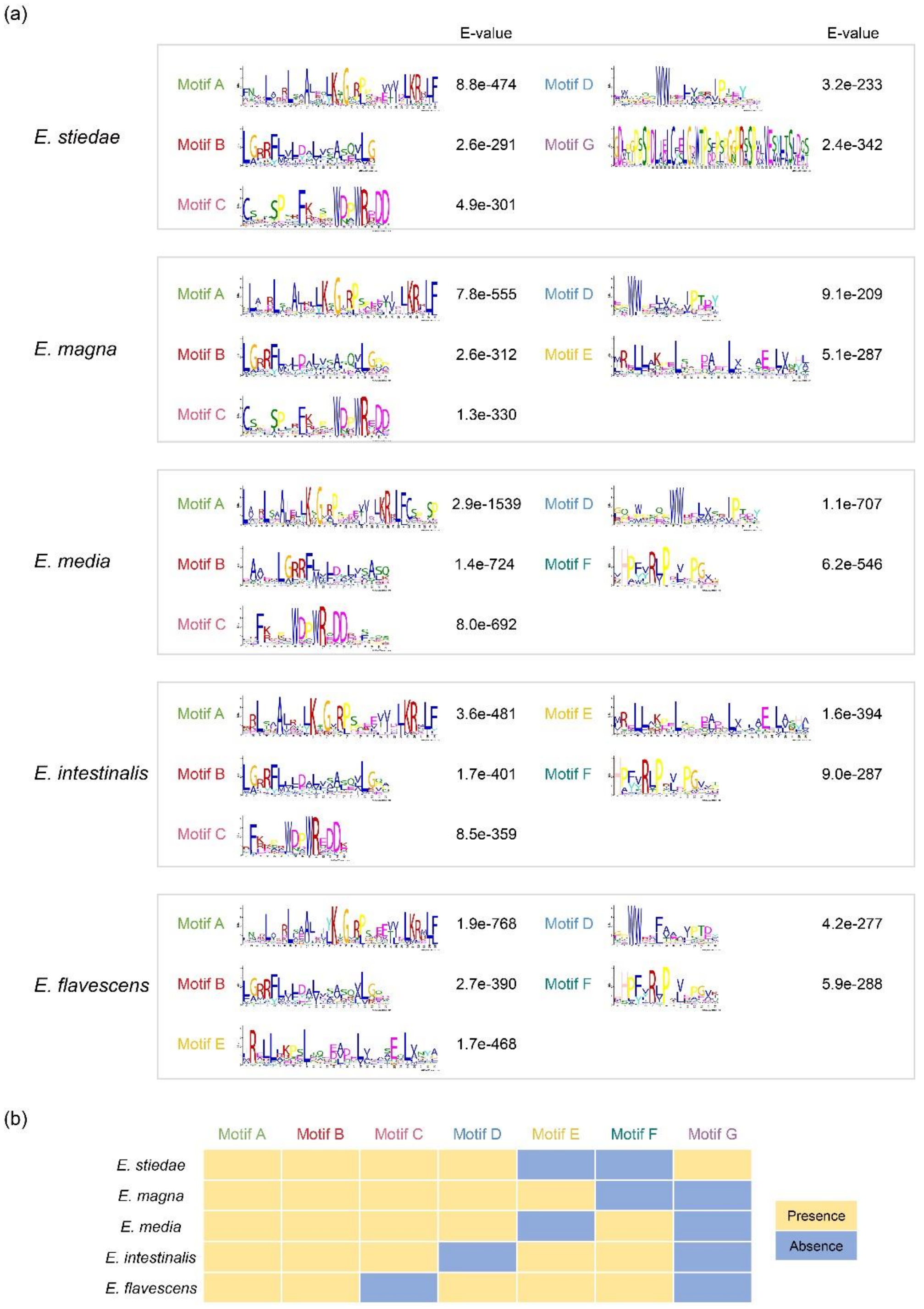

**Fig. S9.** Differences in the transcription of invasion-associated genes between sporulated and unsporulated oocysts of *Eimeria stiedae*. (a) The *p*-values shown in each group are calculated using the Wilcoxon rank-sum analysis of the differences between sporulated and unsporulated oocysts. (b) Transcriptomic levels of genes using the TPM normalization method. The heatmap displays the log2 TPM values for genes in RbE-SAGs, MICs, ROPs, RONs, and GRAs. Genes with red font indicate upregulation in sporulated oocysts, blue font indicate downregulation, and black font represent no significant change.

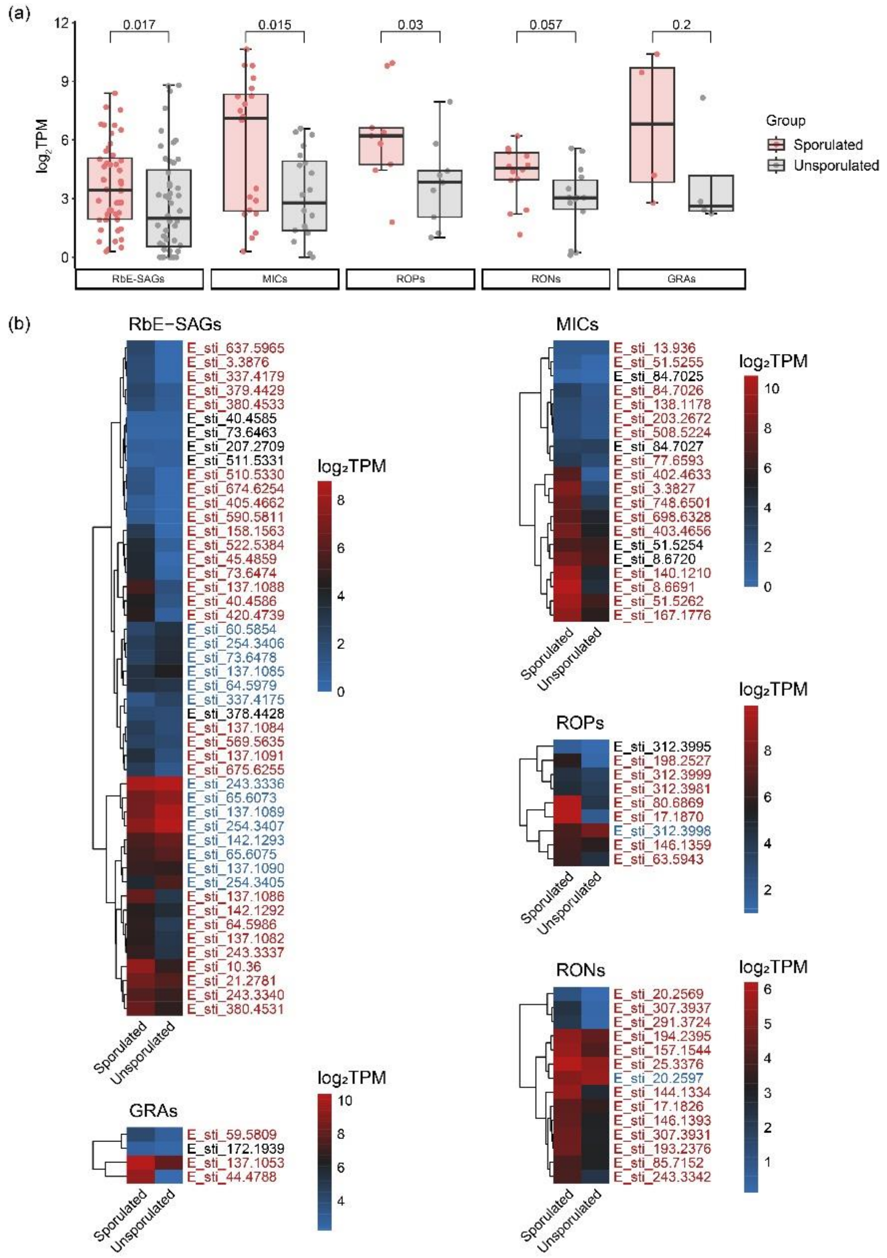

**Fig. S10.** Copy number differences and phylogenetic relationships of RONs among common coccidia. (a) Copy numbers of RONs among common coccidia. RON3, RON4, and RON5 are the major multi-copy RONs in rabbit *Eimeria* spp. (b) Phylogenetic relationships of RONs among five rabbit *Eimeria* and other coccidia.

(a)

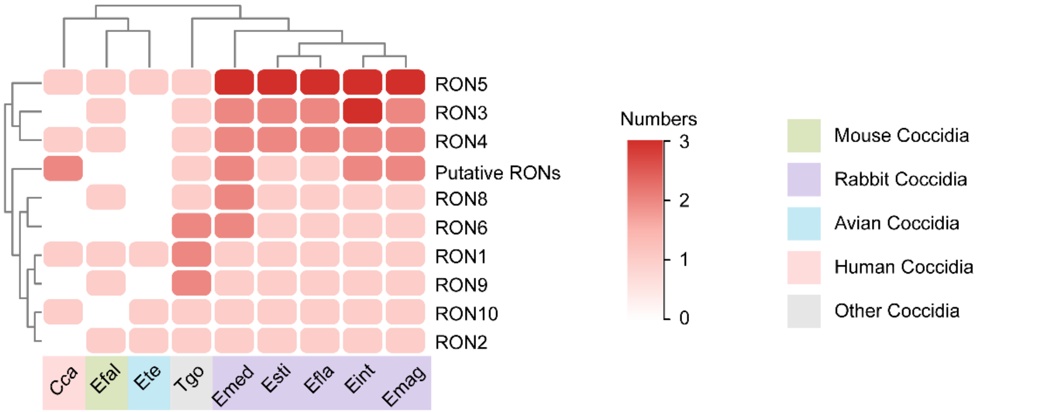


(b)

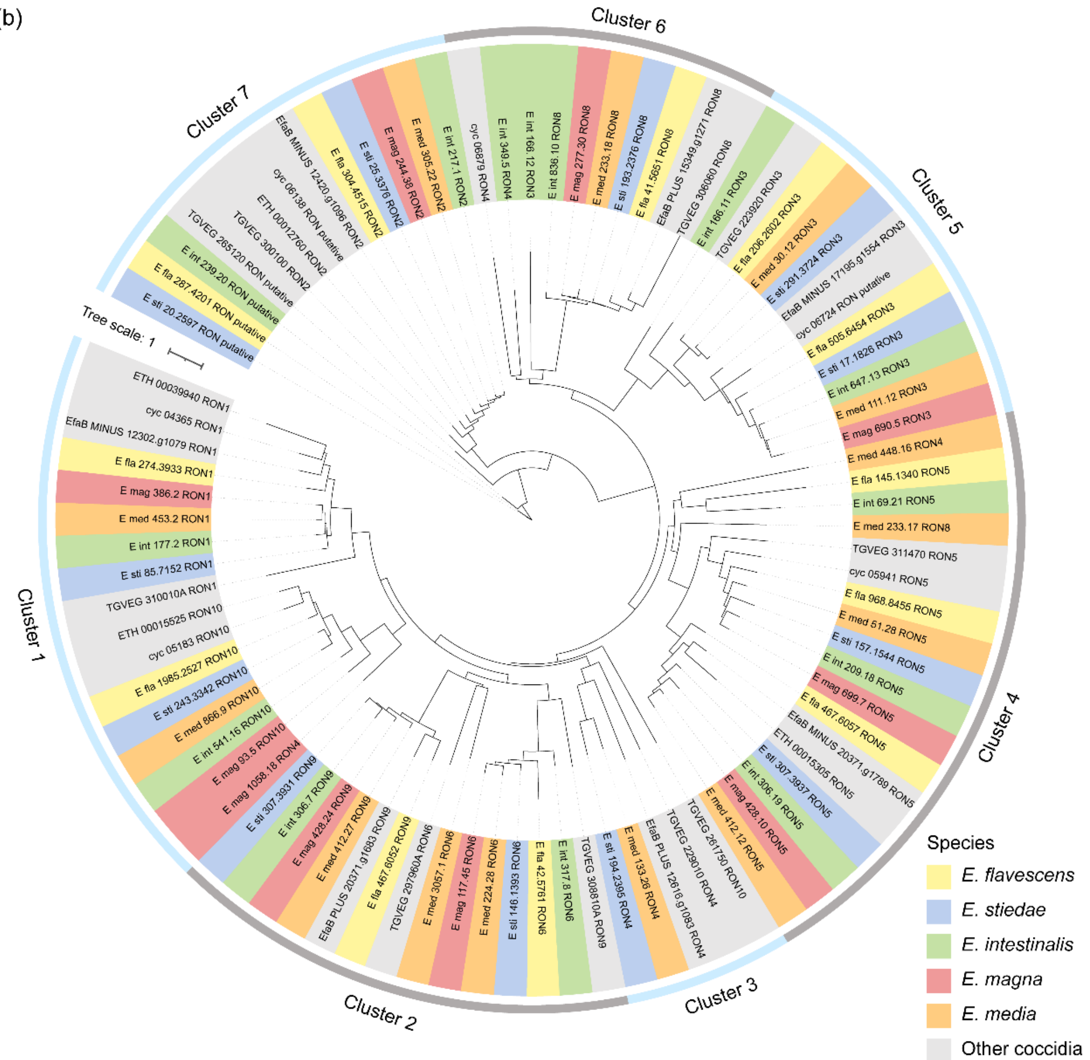

**Fig. S11.** Phylogenetic relationships and functional domains of GRAs in rabbit *Eimeria* spp. (a) Maximum likelihood tree of all identified GRAs. Bootstrap values greater than 50% are shown at the corresponding nodes. (b) Individual maximum likelihood trees of GRA9, GRA10, GRA11, and GRA12. The left panel illustrates the maximum likelihood tree of each type of GRAs, with bootstrap values above 50% indicated at relevant nodes. The right panel presents a schematic representation of gene lengths alongside the mapped positions of their respective domains.

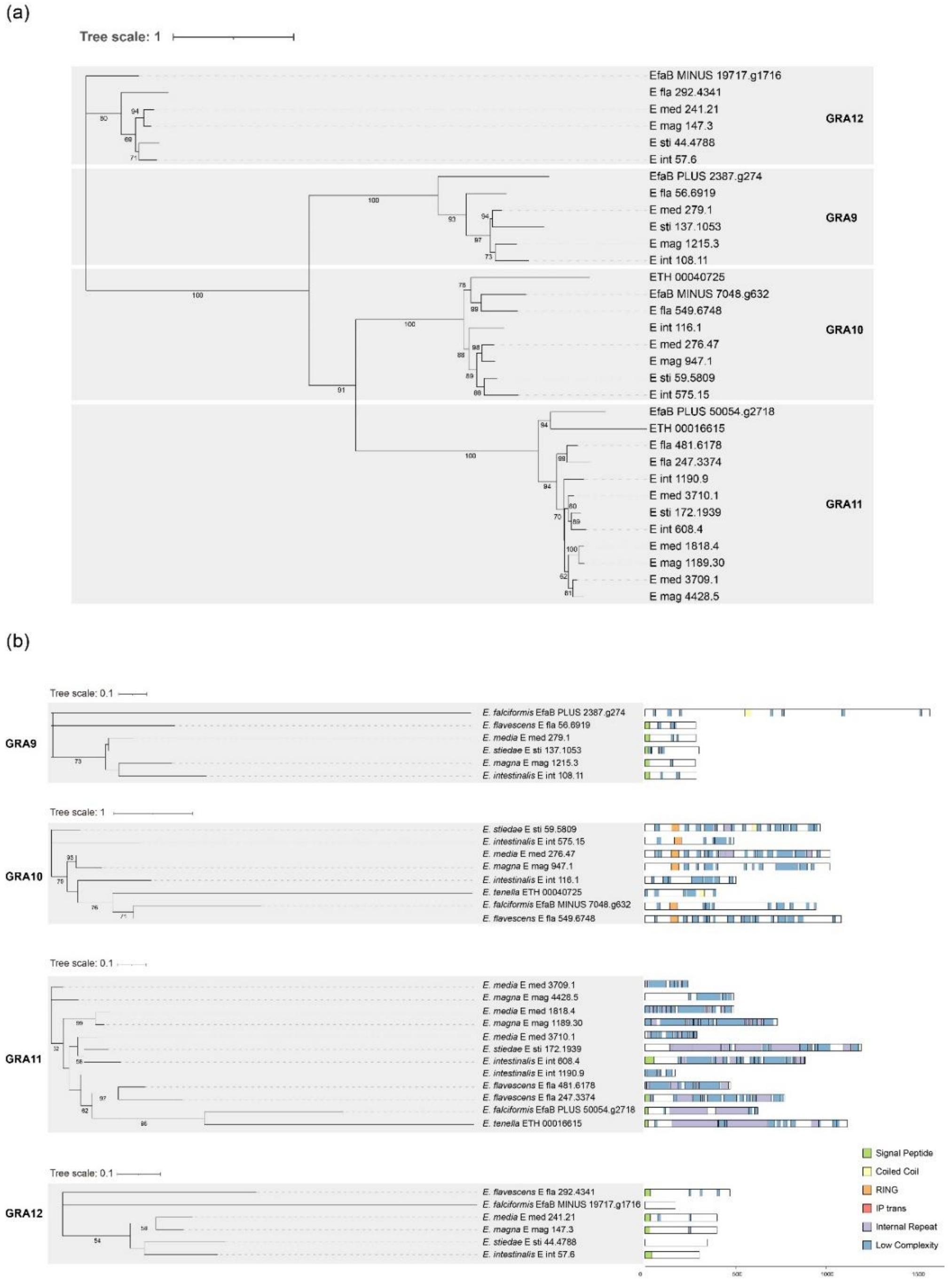

Supplement: Uncited Supplementary Material 1. [file mgen-11-01576-s001.pdf]
